# Pharmacokinetic and pharmacodynamic profile of bendamustine and its metabolites

**DOI:** 10.1007/s00280-015-2727-6

**Published:** 2015-04-01

**Authors:** Mona Darwish, Mary Bond, Edward Hellriegel, Philmore Robertson, James P. Chovan

**Affiliations:** 1 Sci-Med Bridge, LLC, 1916 General Alexander Drive, Malvern, PA 19355 USA; 2 Teva Branded Pharmaceutical Products R&D, Inc., 41 Moores Road, Frazer, PA 19355 USA; 3 Global Nonclinical DMPK, Teva Branded Pharmaceutical Products R&D, Inc., 145 Brandywine Parkway, West Chester, PA 19380 USA

**Keywords:** Chronic lymphocytic leukemia, Indolent B cell non-Hodgkin’s lymphoma, Alkylating agent, Pharmacokinetic profile, Systemic exposure

## Abstract

**Purpose:**

Bendamustine is a unique alkylating agent indicated for the treatment of chronic lymphocytic leukemia and rituximab-refractory, indolent B cell non-Hodgkin’s lymphoma. Despite the extensive experience with bendamustine, its pharmacokinetic profile has only recently been described. This overview summarizes the pharmacokinetics, pharmacokinetic/pharmacodynamic relationships, and drug–drug interactions of bendamustine in adult and pediatric patients with hematologic malignancies.

**Methods:**

A literature search and data on file (including a human mass balance study, pharmacokinetic population analyses in adult and pediatric patients, and modeling analyses) were evaluated for inclusion.

**Results:**

Bendamustine concentrations peak at end of intravenous infusion (~1 h). Subsequent elimination is triphasic, with the intermediate *t*_1/2_ (~40 min) as the effective *t*_1/2_ since the final phase represents <1 % of the area under the curve. Bendamustine is rapidly hydrolyzed to monohydroxy-bendamustine and dihydroxy-bendamustine, which have little or no activity. Cytochrome P450 (CYP) 1A2 oxidation yields the active metabolites γ-hydroxybendamustine and *N*-desmethyl-bendamustine, at low concentrations, which contribute minimally to cytotoxicity. Minor involvement of CYP1A2 in bendamustine elimination suggests a low likelihood of drug–drug interactions with CYP1A2 inhibitors. Systemic exposure to bendamustine 120 mg/m^2^ is comparable between adult and pediatric patients; age, race, and sex have been shown to have no significant effect on systemic exposure in either population. The effect of hepatic/renal impairment on bendamustine pharmacokinetics remains to be elucidated. Higher bendamustine concentrations may be associated with increased probability of nausea or infection. No clear exposure–efficacy response relationship has been observed.

**Conclusions:**

Altogether, the findings support dosing based on body surface area for most patient populations.

## Introduction

Bendamustine hydrochloride is a unique multifunctional alkylating agent with demonstrated clinical activity against various hematologic malignancies when used as monotherapy or in combination with other chemotherapeutic agents [1–6]. Administered as an intravenous infusion, bendamustine is currently indicated for the treatment of chronic lymphocytic leukemia (CLL) and indolent B cell non-Hodgkin’s lymphoma (NHL) that has progressed during or within 6 months of treatment with rituximab or a rituximab-containing regimen [3, 7–9]. The recommended bendamustine dose is 100 mg/m^2^ on days 1 and 2 of a 28-day cycle for CLL and 120 mg/m^2^ on days 1 and 2 of a 21-day cycle for relapsed/refractory NHL [7].

Although its mechanisms of action have not been fully elucidated, bendamustine, like other bifunctional alkylators, crosslinks DNA and produces single- and double-strand breaks; however, in vitro studies have demonstrated that bendamustine causes more extensive and durable breaks than carmustine and cyclophosphamide [10]; has incomplete cross-resistance with other alkylators; and leads to cell death via apoptosis or mitotic catastrophe [1, 10].

**Fig. 1:**
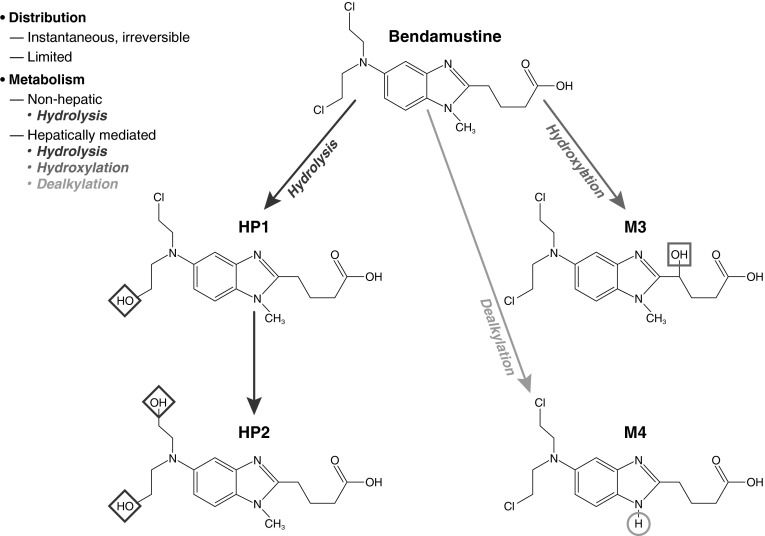
Bendamustine and its main metabolites. Reproduced with permission of ASPET [23]

Chemically, bendamustine is 4-[5-[bis(2-chloroethyl)amino]-1-methyl-benzoimidazol-2-yl]butyric acid hydrochloride [11]. Structurally, bendamustine consists of three moieties: a mechlorethamine group with alkylating properties, a butyric acid side chain that increases water solubility, and a benzimidazole ring that may confer an antimetabolite property (Fig. 1) [11–13]. Bendamustine is primarily metabolized via hydrolysis of its mechlorethamine group into two metabolites with little or no activity: monohydroxy-bendamustine (HP1) and dihydroxy-bendamustine (HP2) [7, 14]. Bendamustine also undergoes phase 1 metabolism via cytochrome P450 (CYP) 1A2-catalyzed oxidative pathways, which result in two active circulating metabolites: γ-hydroxybendamustine (M3), which was previously thought to be β-hydroxybendamustine [15, 16], and *N*-desmethyl-bendamustine (M4) [16]. M3 is produced by γ-oxidation of the butyric acid side chain, and M4 by demethylation of the benzimidazole ring [16].

Despite the extensive clinical experience with bendamustine, its overall pharmacokinetics, pharmacokinetic/pharmacodynamic relationships, and drug–drug interactions have only recently been described. This report provides a comprehensive overview of data characterizing the pharmacokinetics and pharmacokinetic/pharmacodynamic relationships of bendamustine and its circulating active metabolites in adult and pediatric patients with hematologic malignancies. A MEDLINE search for “bendamustine AND (pharmacokinetics OR pharmacodynamics)” was conducted, and the resulting citation list was reviewed for relevant data to be included in this paper. In addition, data on file from the preclinical and clinical development programs, including modeling analyses, were evaluated for possible relevance and inclusion. Specific datasets included a human mass balance study and population pharmacokinetic analyses in adult and pediatric patients.

## Overall pharmacokinetic profile

As shown by a population pharmacokinetic analysis from a phase 3 study in patients with rituximab-refractory, indolent B cell NHL and a human mass balance study [17, 18], bendamustine is metabolized via multiple pathways, has a short effective *t*_1/2_ (~40 min) with the maximum plasma concentration (*C*_max_) typically reached near the end of the infusion period (~1 h), and a low ratio of concentration at 12 h to *C*_max_ (mean 1:25,000). Thus, although the pharmacokinetics of multiple-dose administration of bendamustine have not been studied, dose accumulation is not expected with the standard dosing schedule of two consecutive days in a 21- or 28-day cycle. Therefore, the single-dose pharmacokinetic profile is considered representative of the multiple-dose pharmacokinetic profile [17, 18].

### Distribution

In vitro, approximately 95 % of bendamustine is protein bound (mostly to albumin) [7, 19]. Data suggest that bendamustine is not likely to displace or be displaced by highly protein-bound drugs [19].

In mice administered [^14^C]bendamustine, tissue levels of radioactivity were substantially higher in the liver and kidneys—two highly perfused organs involved in the clearance of bendamustine and its metabolites—than other tissues [20]. In human adults, the mean steady-state volume of distribution for bendamustine was estimated in two studies to be ~25 L [17] and 20 L [18], which, consistent with murine radioactivity findings, indicates that the drug is mainly confined to the extracellular fluid and not extensively distributed to tissues [7]. This pattern of distribution is also consistent with the effectiveness of bendamustine in inhibiting the growth of lymphoma tumors in xenograft models [21, 22].

### Metabolism

Bendamustine is primarily hydrolyzed (mainly nonenzymatically) to the much less active HP1 and HP2 [7, 14, 23]. Bendamustine is also metabolized by CYP1A2 enzymes to active M3 and M4 [16, 23], which reach their maximum concentrations at or near the same time as the parent drug [17]. The potency of M3 is approximately equivalent to that of bendamustine, although that of M4 is five- to ten-fold less than that of the parent drug [16]. Because plasma concentrations of M3 and M4 are only 1/10 and 1/100 that of bendamustine, respectively [17], the contribution of M3 and M4 to the therapeutic activity of bendamustine would be expected to be minimal [7, 16, 18]. In addition, these low plasma concentrations suggest that hepatic metabolism via the CYP1A2 oxidative pathways plays a minor role [23].

Most metabolic pathways for bendamustine were originally observed in rats, such as hydrolysis (the primary metabolic process), cysteine conjugation of the mechlorethamine moiety, and *N*-demethylation and γ-hydroxylation of the benzimidazole and butyric acid moieties, respectively [24].

Analyses of urine samples from the human mass balance study were conducted to more fully characterize the metabolite profile of bendamustine [23]. Urine samples collected 2 h after the start of [^14^C]bendamustine infusion were used because they were found to have the highest concentrations of radioactivity. Radiochromatograms of these samples revealed a total of 25 bendamustine-related compounds as well as bendamustine. The majority of the metabolites that were detected were present only during the early periods of sample collection. [^14^C]Bendamustine-derived materials that were detectable in late urine samples (up to 168 h after the infusion) included products of dihydrolysis and cysteine conjugation of bendamustine and γ-hydroxybendamustine.

Although a few new metabolic products were detected in the human mass balance study that had not been observed in rats, those products were largely found to represent adducts that formed by reaction of bendamustine with endogenous compounds in the urine, e.g., phosphate, creatinine, and uric acid. These reactions were reproduced in vitro by incubation of bendamustine with urine from a drug-naïve subject.

Overall, these findings indicate that the metabolic elimination of bendamustine is qualitatively the same in humans and rats [23].

### Elimination

The plasma concentration versus time profile of bendamustine declines in a polyphasic manner in the phase 3 population pharmacokinetic study and the human mass balance study, with an effective *t*_1/2_ of ~40 min [17, 18]. The active metabolites M3 and M4 have a *t*_1/2_ of similar magnitude [7, 16, 18].

In the population pharmacokinetic model, bendamustine declined from *C*_max_ in a triphasic manner, with an intermediate elimination *t*_1/2_ of ~40 min and a slow terminal *t*_1/2_ of ~110 h [17]. The last phase was only measured after the introduction of advanced technology that allowed for detection of very low concentrations of bendamustine. Because the terminal elimination phase represents a small portion of the overall systemic exposure of bendamustine (<1 %), the intermediate *t*_1/2_ is considered the effective *t*_1/2_ of bendamustine [17]. The human mass balance study showed a similar rapid initial distribution phase and effective *t*_1/2_. Although the slow terminal phase was not observed in the mass balance study, this was probably due to the fivefold higher lower limit of quantitation for the method used [18].

Concentrations of M3 and M4 declined in biphasic and monophasic manners with elimination *t*_1/2_ of ~3 and ~0.7 h (and plasma levels generally undetectable by ~13 and ~5 h), respectively, in the population pharmacokinetic model [17]. In the human mass balance study, the elimination *t*_1/2_ for M3 and M4 were similar (~1.6 and ~0.5 h) [18].

During the 168-h period, after the infusion of [^14^C]bendamustine in the human mass balance study, the mean total recovery of radioactivity in excreta was 76 %, of which ~50 % was recovered in urine and ~25 % in feces. In 24 h after the start of the infusion, ~3 % of the dose was recovered in urine as bendamustine, <1 % each was recovered as M3 and M4, and ~5 % was recovered as HP2 [18, 23]. The high and persistent levels of total radioactivity in urine (~36 % of radiochemical dose after 24 h) and plasma (mean terminal *t*_1/2_ of 197 h after 168 h) compared with those of bendamustine, M3, M4, and HP2 are not uncommon for alkylating agents [25] and indicate the presence of additional longer-lived adducts. As shown in the human mass balance study, bendamustine is extensively metabolized, primarily via non-enzymatic hydrolysis. Renal or hepatic impairment would, therefore, not be expected to have an important effect on the systemic exposure to bendamustine, due to the short *t*_1/2_, dosing schedule, primary metabolic pathways, and the very low renal excretion of bendamustine [18, 23].

## Comparison of adult and pediatric pharmacokinetic profiles

At present, bendamustine is not indicated for the treatment of pediatric acute leukemia, but the pharmacokinetic profiles between adult and pediatric populations are similar.

Bendamustine monotherapy was recently investigated in an international, open-label, single-arm, multicenter, phase 1/2 study of heavily pretreated pediatric patients aged 1–20 years with relapsed/refractory acute lymphoblastic leukemia (ALL) or acute myeloid leukemia (AML) [26]. Secondary study objectives included determining the pediatric pharmacokinetic profile of bendamustine relative to the adult pharmacokinetic profile [27].

Systemic exposure to bendamustine 120 mg/m^2^ in children was similar to that previously reported in the phase 3 adult NHL study [17, 27]. In the 120 mg/m^2^ pediatric group (*n* = 38), mean *C*_max_ was 6806 ng/mL (mean *t*_max_, 1.1 h) and mean area under the curve (AUC)_0–24_ was 8240 ng h/mL, compared with a mean *C*_max_ of 5746 ng/mL and mean AUC_0–24_ of 7121 ng h/mL in adults (*n* = 78) [26, 27].

As with adults, the *C*_max_ of bendamustine 120 mg/m^2^ in pediatric patients was reached by the end of infusion (~1 h). The pediatric pharmacokinetic profile for bendamustine showed a very rapid distribution phase after the peak plasma concentration, followed by a somewhat slower drug elimination phase. The third phase decline, which has been observed in adults and represents <1 % of overall AUC, could not be adequately shown in the pediatric study due to limited sampling (no samples were collected in the 12–24 h timeframe, with few samples at later time points) [27].

## Bendamustine dosing paradigm

Bendamustine dosing is based on body surface area (BSA) to reduce interindividual variability in drug concentrations and to achieve comparable systemic exposure across patients. Recent data confirm the appropriateness of a BSA-based dosing scheme for bendamustine [27].

**Fig. 2:**
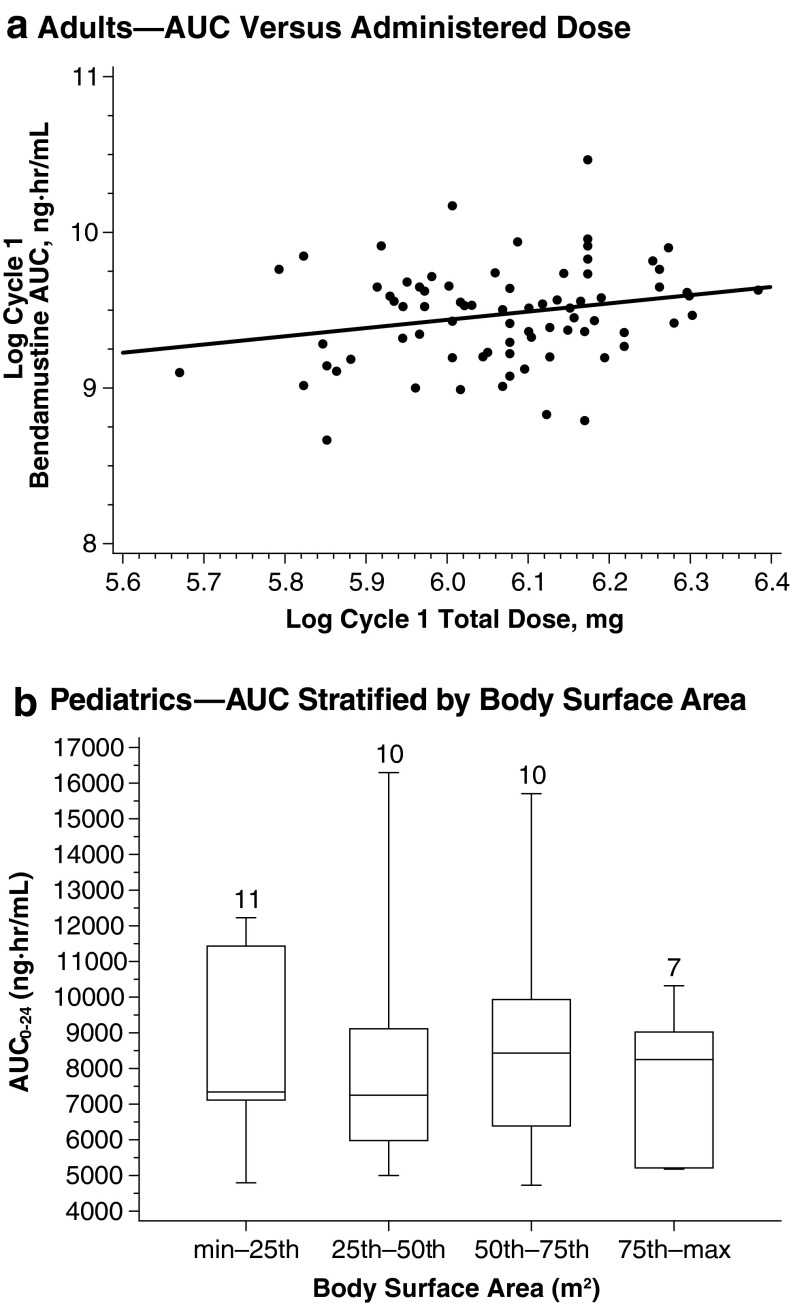
Effect of body surface area on systemic exposure. **a** The *line* represents a linear regression. **b** *Boxes* are 25th, 50th, and 75th percentiles; whiskers are 5th and 95th percentiles. The *numbers* above the *box* represent the number of patients. Pediatrics panel: adapted with permission of Informa Healthcare [27]

A population pharmacokinetic analysis in 43 pediatric patients with acute leukemia who received bendamustine (120 mg/m^2^, *n* = 38; 90 mg/m^2^, *n* = 5) in the phase 1/2 pediatric study demonstrated comparable systemic exposure and little difference (<15 %) in median bendamustine AUC and *C*_max_ across BSA quartiles, despite a wide range of BSAs (0.49–1.86 mg/m^2^) (Fig. 2) [27]. No dose-limiting toxicities were reported [26]. Because systemic exposure to bendamustine 120 mg/m^2^ was similar between adult and pediatric patients—with mean AUC_0–24_ and *C*_max_ values <16 % higher in the pediatric patients population—higher doses were not assessed in the pediatric study, per protocol [17, 27].

## Effect of selected covariates on the pharmacokinetics of bendamustine in adult and pediatric patients

The potential impact of age, sex, race, and hepatic or renal impairment on the pharmacokinetics of bendamustine has been assessed in both adult and pediatric patients using population pharmacokinetic analysis. Available evidence does not suggest significant differences based on age, sex, or race. Mild hepatic and renal impairment did not show significant effects on the pharmacokinetics of bendamustine; however, some differences in systemic exposure cannot be ruled out.

Within the population pharmacokinetic analysis, covariates were assessed using forward selection and backward elimination procedures [17]. A model was created and individual concentration–time profiles and pharmacokinetic parameters were generated using the Bayesian approach. Individual systemic exposures (expressed as *C*_max_ and AUC) were then compared between adult and pediatric patients [17, 27]. Because data from the adult and pediatric population pharmacokinetic analyses were limited for certain covariates, definitive conclusions regarding the potential impact of those covariates could not be drawn.

### Effect of age on systemic exposure to bendamustine

Overall data from adult and pediatric studies provide evidence that age is not an important determinant of systemic exposure to bendamustine [17, 27].

#### Adult patients

**Fig. 3:**
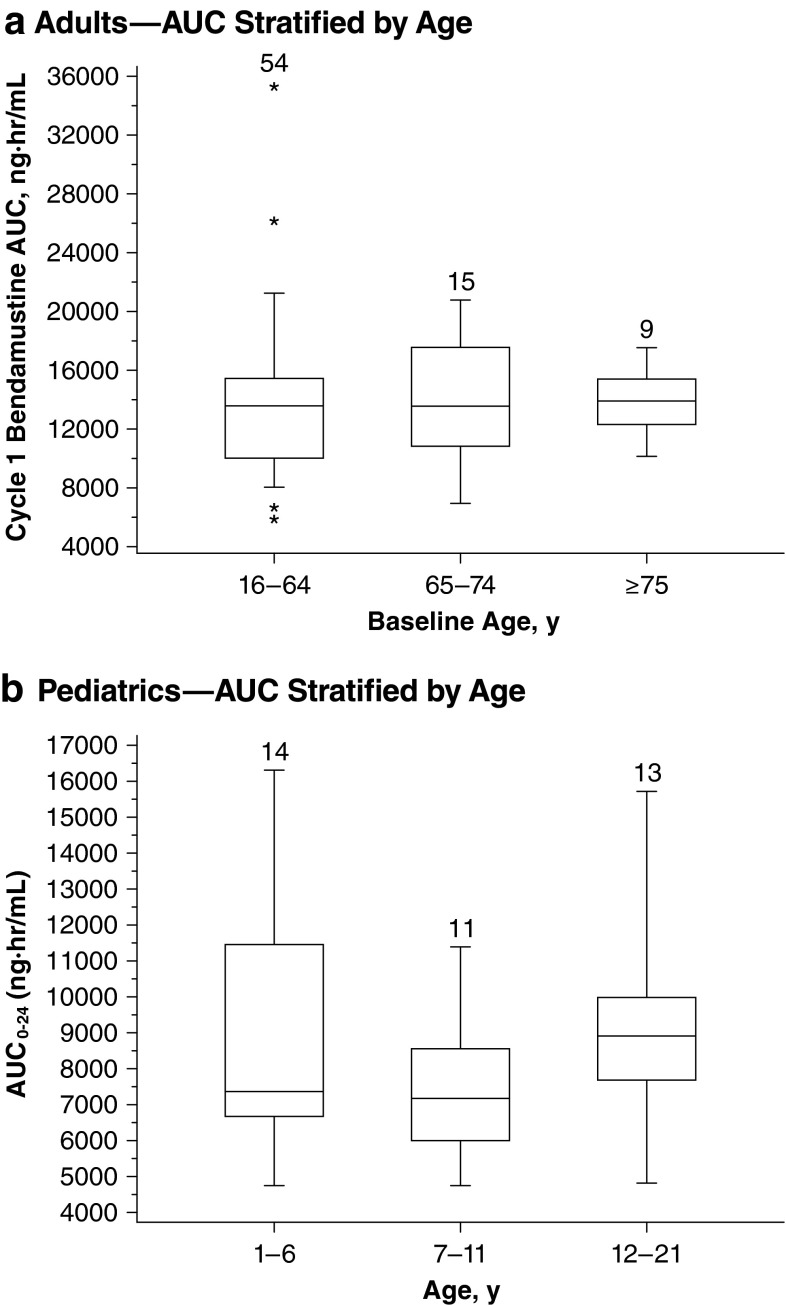
Effect of age on systemic exposure. *Boxes* are 25th, 50th, and 75th percentiles; whiskers are 5th and 95th percentiles. *Asterisks* are data points outside this range. The *numbers* above the *box* represent the number of patients. Pediatrics panel: adapted with permission of Informa Healthcare [27]

In the phase 3 study in adults with NHL, the median bendamustine *C*_max_ and AUC showed little difference (<6 %) among three age groups (16–64, 65–74, and ≥75 years) following bendamustine 120 mg/m^2^ (Fig. 3). In a model based on that study, the predicted means for *C*_max_ and AUC_0–24_ across the entire age range were 5746 ng/mL and 7121 ng h/mL, respectively [17, 27].

#### Pediatric patients

Similarly, median bendamustine AUC and *C*_max_ varied by <20 % across all age groups (1–6, 7–11, and 12–21 years) in the pediatric study (Fig. 3) [27]. Furthermore, mean *C*_max_ and AUC_0–24_ across the entire age range in the pediatric study were 6806 ng/mL and 8240 ng h/mL, respectively, which were comparable to those of the adult population [27].

### Effect of sex on systemic exposure to bendamustine

The sex of adult or pediatric patients has been shown to have no significant effect on systemic exposure to bendamustine.

#### Adult patients

In the phase 3 adult NHL study, there were no notable differences between median bendamustine AUC and *C*_max_ for men and women, which were within 2 % of each other [17, 27].

#### Pediatric patients

Sex differences in median bendamustine AUC and *C*_max_ levels in the pediatric population pharmacokinetic analysis did not meet the prespecified significance level in the population analysis. However, the levels were lower by 26 and 16 %, respectively, in male compared with female patients [27]. The reason for the apparent higher exposure in female patients remains unknown [27].

### Effect of race on systemic exposure to bendamustine

Race appears to have no significant effect on systemic exposure to bendamustine.

#### Adult patients

There were too few non-Caucasian patients in the phase 3 NHL study to draw any conclusions regarding the influence of race on bendamustine systemic exposure in adults [17]. However, there was no evidence of any differences in pharmacokinetic properties among the different race groups, including black (*n* = 5), Asian (*n* = 1), and Hispanic (*n* = 1) patients. The pharmacokinetic profiles of 12 Japanese patients with relapsed/refractory NHL or mantle cell lymphoma who received bendamustine 120 mg/m^2^ alone (*n* = 6; *C*_max_ 8.6 μg/mL, AUC_0–t_ 10.2 μg h/mL) or in combination with rituximab (*n* = 6; *C*_max_ 5.4 μg/mL, AUC_0–t_ 6.1 μg h/mL) were demonstrated to be similar to that of patients in the phase 3 adult NHL study (*n* = 78; *C*_max_ 5.8 μg/mL, AUC_0–t_ 13.6 μg h/mL) [17, 28, 29].

#### Pediatric patients

In the pediatric study of bendamustine 120 mg/m^2^, median AUC and *C*_max_ values in Caucasians (*n* = 20) and Asians (*n* = 11) were within <5 % of each other [27]. Although bendamustine systemic exposure was ≤30 % lower in non-Caucasian/non-Asian patients (*n* = 7, most of whom were black or Hispanic) than in Caucasian and Asian patients, the difference did not meet the prespecified level of significance for bendamustine pharmacokinetic parameters.

### Effect of hepatic impairment on systemic exposure to bendamustine

The effect of hepatic impairment on the pharmacokinetics of bendamustine remains to be fully elucidated. Although no significant change in bendamustine clearance has been noted in patients with mild hepatic impairment [7, 17], some differences in bendamustine systemic exposure in this population cannot be ruled out. Due to limited data, the current recommendation is for bendamustine to be used with caution in patients with mild hepatic impairment and not to be used in patients with moderate hepatic impairment (aspartate aminotransferase [AST] or alanine aminotransferase [ALT] 2.5–10 × upper limit of normal [ULN] and total bilirubin 1.5–3 × ULN) or severe hepatic impairment (total bilirubin >3 × ULN) [7].

#### Adult patients

**Fig. 4:**
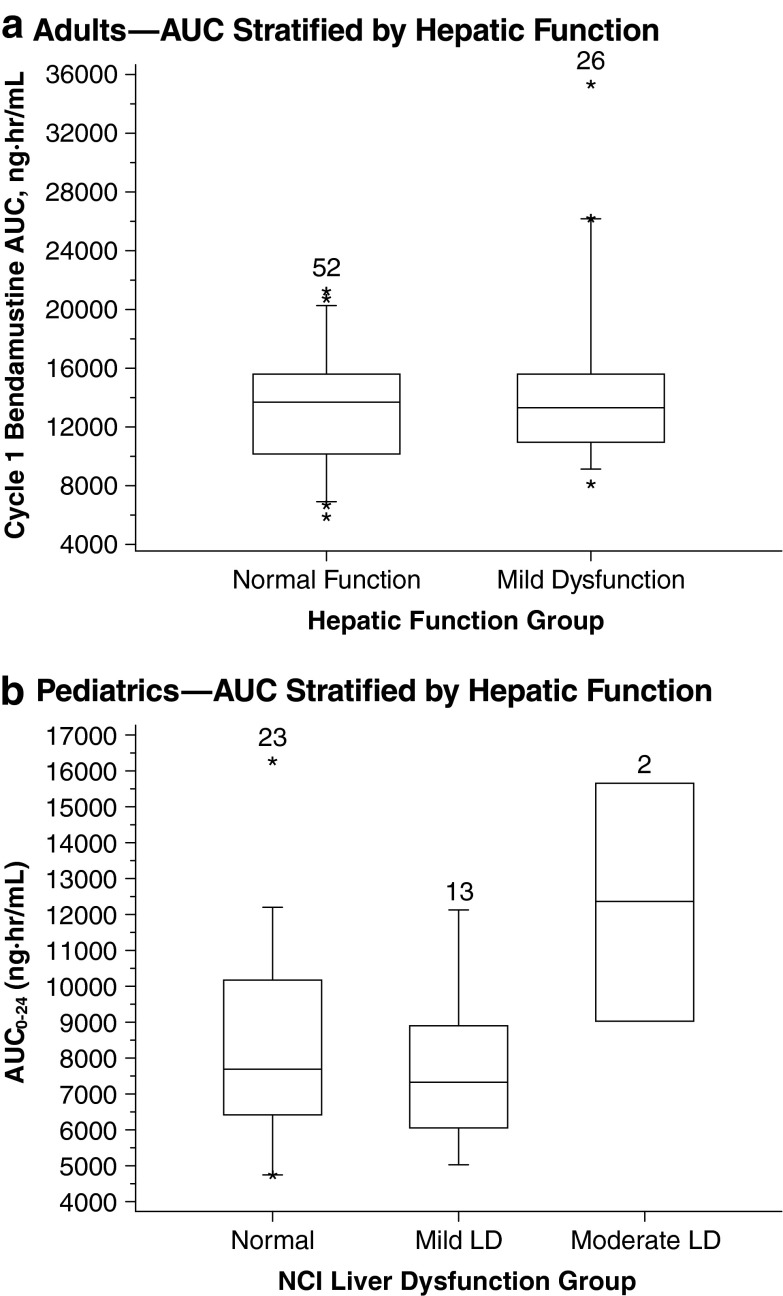
Effect of hepatic impairment on systemic exposure. *Boxes* are 25th, 50th, and 75th percentiles; whiskers are 5th and 95th percentiles. *Asterisks* are data points outside this range. The *numbers* above the *box* represent the number of patients. Pediatrics panel: adapted with permission of Informa Healthcare [27]

In the adult NHL phase 3 study, the pharmacokinetic profile of bendamustine in 26 patients with mild hepatic impairment (defined as total bilirubin ≤ULN, AST ≥ ULN to 2.5 × ULN, and/or alkaline phosphatase ≥ULN to 5.0 × ULN) was not substantially different from that in 52 patients with normal function (Fig. 4) [7, 17]. In addition, bendamustine tolerability was found to be adequate in a pilot study of six patients with advanced bile duct cancer and substantial hepatic dysfunction (bilirubin ≤ 3 × UNL) [30]. Furthermore, two patients with severe liver impairment and aggressive NHL were recently reported to have been successfully treated with bendamustine and rituximab [31]. Their total serum bilirubin levels were 10 × ULN, but improved greatly after treatment; mild-to-moderate increases in ALT and AST levels in both cases also improved following treatment. The liver impairment in both patients was considered obstructive rather than functional [31].

#### Pediatric patients

In the pediatric population pharmacokinetic analysis, a difference of ≤5 % was observed in the median bendamustine AUC_0–24_ and *C*_max_ in the 23 patients with normal hepatic function and the 13 patients with mild hepatic impairment. Patients with moderate hepatic impairment (*n* = 2) had higher bendamustine systemic exposure (Fig. 4) [27].

### Effect of renal impairment on systemic exposure to bendamustine

The effect of renal impairment on the pharmacokinetics of bendamustine remains to be fully elucidated. Although no significant change in bendamustine clearance has been noted in patients with mild-to-moderate renal impairment [7, 17], some differences in bendamustine systemic exposure in this population cannot be ruled out. Given that only ~3 % of the bendamustine dose is eliminated renally, renal impairment would be unlikely to have a substantive effect on bendamustine systemic exposure [18, 32]. However, due to limited data, the current recommendation is for bendamustine to be used with caution in patients with mild-to-moderate renal impairment and not to be used in patients with creatinine clearance (CrCL) <40 mL/min [7].

#### Adult patients

**Fig. 5:**
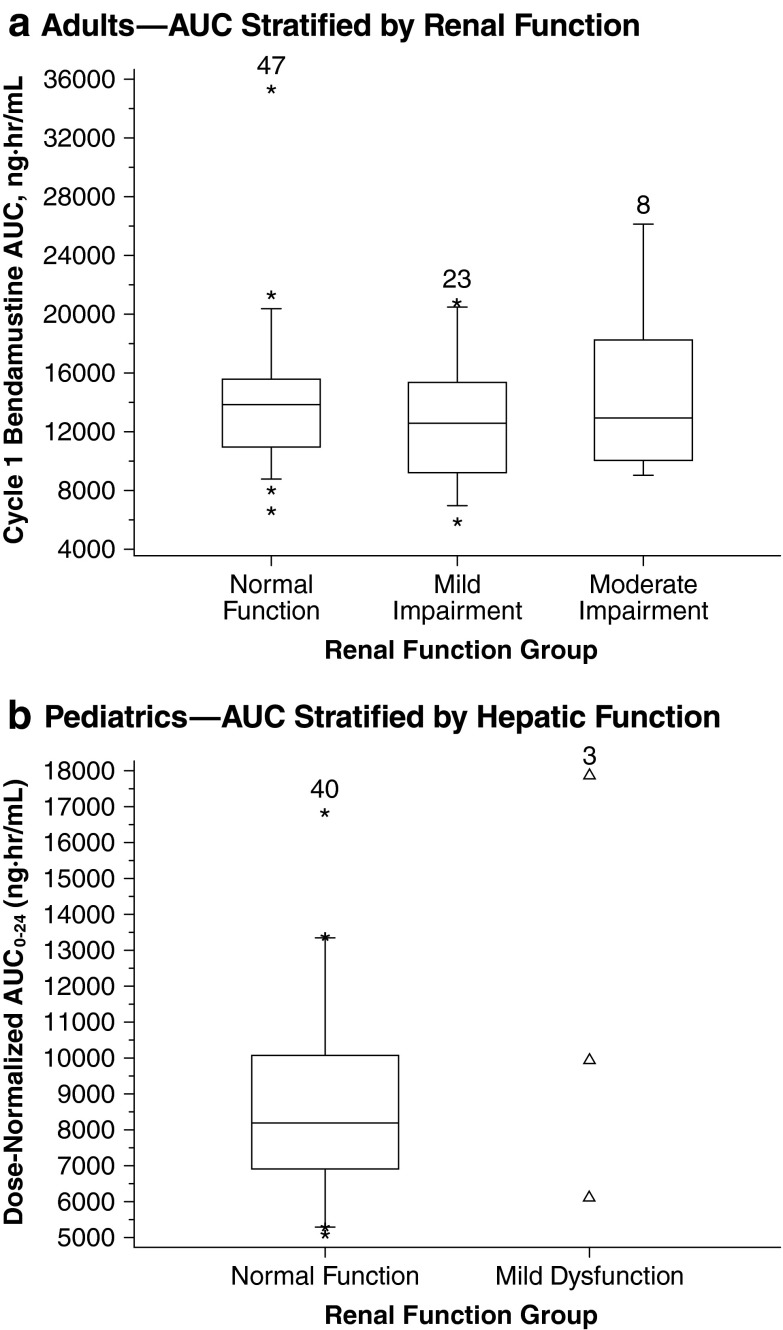
Effect of renal impairment on systemic exposure. *Boxes* are 25th, 50th, and 75th percentiles; whiskers are 5th and 95th percentiles. *Asterisks* are data points outside this range. *Triangles* show individual data points for patients with mild renal dysfunction. The *numbers* above the *box* represent the number of patients. Pediatrics panel: adapted with permission of Informa Healthcare [27]

In the adult phase 3 NHL study, there was no meaningful difference in the pharmacokinetics of bendamustine among the 31 patients with mild or moderate renal impairment (CrCL, 30–80 mL/min) [7, 17] and the 47 patients with normal renal function (Fig. 5). In addition, a myeloma study showed no differences in the plasma kinetics of bendamustine or its metabolites between patients with normal renal function (*n* = 12) and those with renal insufficiency (*n* = 12, including 5 who were under continuous hemodialysis), with only a moderate increase in the frequency of myelotoxicity observed in the renally impaired group, and no dose reductions were required [32]. A retrospective safety assessment in NHL and CLL of 104 renally impaired patients (CrCL of <40 mL/min) and 836 patients with CrCL ≥40 mL/min showed no significant differences in laboratory toxicities between the CrCL groups [33]. Renally impaired patients were found to have a twofold increase in the risk of two evaluated grade 3–4 adverse events compared with patients who had a CrCL ≥40 mL/min: increased blood urea nitrogen for CLL and NHL together (*P* = 0.02), and thrombocytopenia in a subanalysis of NHL patients with a CrCL <40 mL/min versus those with NHL and a CrCL ≥60 mL/min (*P* = 0.025) [33]. Likewise, in two prospective clinical studies [34, 35] and one retrospective study [36] of myeloma patients with moderate-to-severe renal impairment or renal failure/dialysis, bendamustine in combination with other drugs (prednisone and bortezomib, or thalidomide and dexamethasone) was well tolerated.

#### Pediatric patients

In pediatric patients, no differences in dose-normalized bendamustine pharmacokinetics were observed between those with mild renal impairment (*n* = 3), as defined by glomerular filtration rate per National Kidney Foundation 2002 age-based guidelines [37], and normal renal function (*n* = 40) (Fig. 5) [27].

## Potential for CYP interactions with bendamustine

Because bendamustine is primarily biotransformed via hydrolysis [7, 14, 23], there is limited potential for direct drug interaction.

Based on in vitro data, bendamustine has a low potential to affect drug metabolism via human cytochrome P450 enzymes. At concentrations up to 200 μM, bendamustine did not inhibit the metabolism of substrates specific for isoenzymes CYP1A2, CYP2C9/10, CYP2D6, CYP2E1, or CYP3A4/5, and at up to 100 μM, it showed no potential for induction of CYP enzymes [7].

**Fig. 6:**
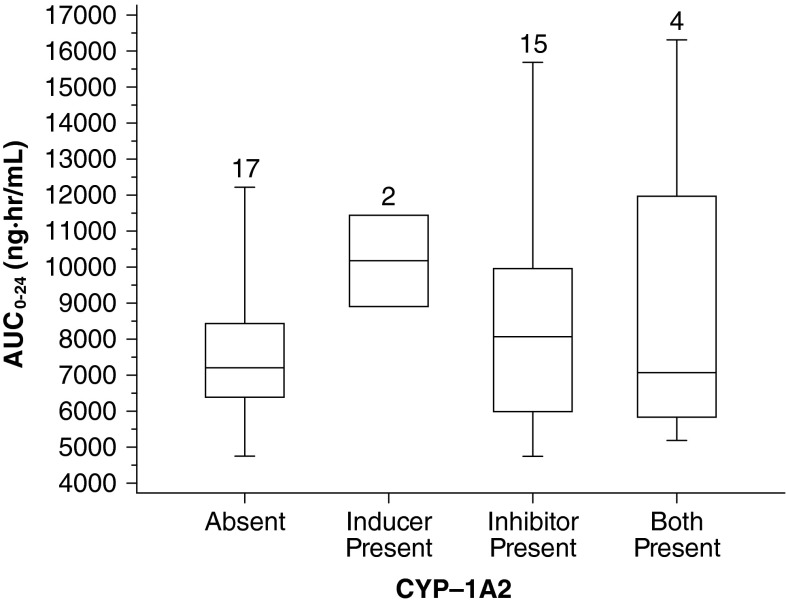
Pharmacokinetics in presence or absence of CYP1A2 inhibitors/inducers. *Boxes* are 25th, 50th, and 75th percentiles; whiskers are 5th and 95th percentiles. The *numbers* above the *box* represent the number of patients. Adapted with permission of Informa Healthcare [27]

However, the M3 and M4 metabolites, both of which make little contribution to the cytotoxicity of bendamustine [7, 16, 18], are formed by CYP1A2 [16]. As a result, systemic exposure to bendamustine in the presence and absence of CYP1A2 inhibitors and inducers was evaluated. A comparison between the observed bendamustine concentration–time profile following coadministration with a CYP1A2 inhibitor (e.g., allopurinol, famotidine, ranitidine, or ciprofloxacin) in 15 patients or with a CYP1A2 inducer in two patients was similar to that following administration without a CYP1A2 inhibitor/inducer, which confirms that oxidative metabolism by CYP1A2 is a relatively minor contributor to the elimination of bendamustine (Fig. 6).

## Bendamustine exposure–response relationship

Data suggest that *C*_max_ is an essential component of the activity of bendamustine.

The excess of B cells associated with CLL is caused by a decrease in apoptosis rather than an increase in cell proliferation [38–41]. In vitro, bendamustine has been shown to induce apoptosis in a dose- and time-dependent manner in B-CLL lymphocytes, and elevated plasma concentrations seem to be more relevant than prolonged exposure [42]. Ex vivo studies conducted to assess the effect of bendamustine on leukemic cells in CLL have shown that the median lethal dose (LD_50_) of bendamustine is 4.3 μg/mL in cells from previously treated patients and 7.4 μg/mL in cells from previously untreated patients [42]. In the adult relapsed/refractory NHL phase 3 study, bendamustine 120 mg/m^2^ resulted in a peak exposure of ~6 μg/mL (within the LD_50_ range) [17].

### Adult patients with NHL

**Fig. 7:**
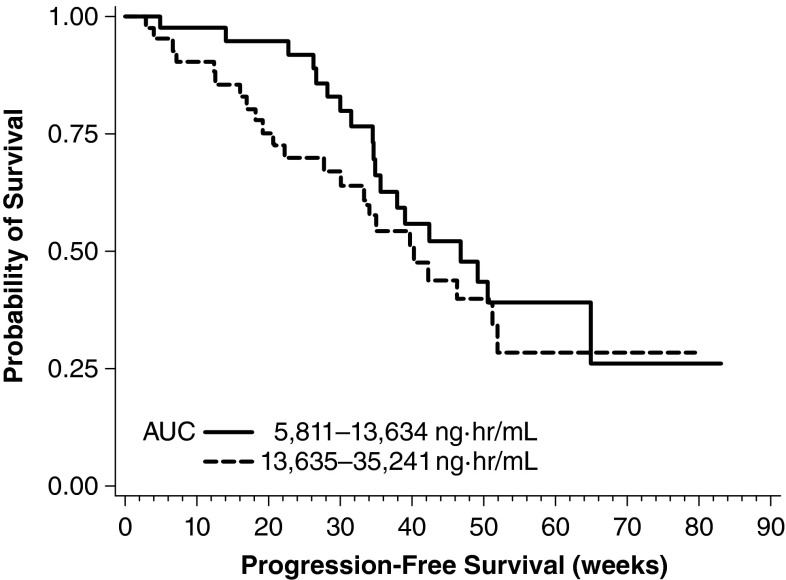
Kaplan–Meier plot of PFS, stratified by median bendamustine AUC. With kind permission from Springer Science+Business Media: Figure 5 [17]

The pharmacokinetic profile of bendamustine and exposure–response relationships were described in 80 patients in the adult NHL phase 3 trial who received bendamustine 120 mg/m^2^ [17]. Eighty-five percent of the patients had at least a partial response following treatment with bendamustine, but there were no significant associations between any of the measures of exposure (e.g., bendamustine AUC and *C*_max_) and treatment response or duration of response. A separate trend was noted in progression-free survival based on bendamustine AUC value above and below the median value (*P* = 0.3025; Fig. 7).

### Pediatric patients with acute leukemia

The population pharmacokinetic and pharmacodynamic analysis in 43 heavily pretreated pediatric patients explored the exposure–response relationship in the 38 patients who received single-agent bendamustine at 120 mg/m^2^ and were evaluable for efficacy in this dose-ranging/safety study [27]. Of the 22 patients receiving 120 mg/m^2^ with a defined best overall response, 9 % achieved a partial response, 32 % had stable disease, and 59 % had progressive disease. The remaining five patients received bendamustine 90 mg/m^2^, two of whom achieved a complete response (both with ALL) [26]. Although no clear exposure–response relationship was observed in the efficacy analysis [27], two ALL patients with partial response as their best overall response had slightly higher bendamustine AUC and *C*_max_ values than the median systemic exposures of patients with stable or progressive disease [26]. No patients with AML had a response. Furthermore, the median bendamustine AUC and *C*_max_ for the 16 AML patients were 17 and 16 % lower, respectively, than for the 22 ALL patients. Response data for the study population suggest that single-agent bendamustine has some activity in heavily pretreated patients with relapsed and refractory ALL, but not in AML [27].

## Bendamustine safety–exposure relationships

The relationship between bendamustine systemic exposure and specific adverse events during treatment was evaluated in the adult and pediatric population pharmacokinetic analyses [17, 27]. Significant correlations were observed only for nausea in the adult population and infection (e.g., aspergillosis, paronychia, sinusitis, and staphylococcal infection) in the pediatric population. It should be noted that although nausea was evaluated in both studies, it was not shown to be associated with bendamustine systemic exposure in pediatric patients.

### Adult patients with NHL

The correlation between exposure and safety was reported in 80 patients who received bendamustine 120 mg/m^2^ in the adult NHL phase 3 trial [17]. Among the five safety endpoints of interest (i.e., neutropenia, thrombocytopenia, nausea, vomiting, and fatigue), only nausea was found to be significantly correlated with bendamustine *C*_max_ (*P* = 0.013), with the probability of nausea increasing along with bendamustine *C*_max_.

The high rate of prophylactic antiemetic use (80 %) prevented the statistical analysis of the influence of their use on the association between bendamustine exposure and nausea [17].

### Pediatric patients with acute leukemia

In the population pharmacokinetic and pharmacodynamic safety analysis in 43 pediatric patients, infection was the only adverse event that was shown to be significantly correlated with bendamustine *C*_max_ (*P* < 0.5). The most common infections were aspergillosis (*n* = 2, 120 mg/m^2^), sinusitis (*n* = 1 each, 90 and 120 mg/m^2^), and staphylococcal infection (*n* = 2, 120 mg/m^2^). The most common grade 3/4 infections were the two patients with aspergillosis and two with staphylococcal infection. As expected, the risk of developing an infection was greater in the absence of prophylactic antibiotic use (*n* = 9). No other exposure measures were a significant predictor of developing an infection [27].

## Potential for drug–drug interactions between bendamustine and monoclonal antibodies

Although not currently approved as combination therapy, bendamustine has demonstrated improved overall response rates and progression-free survival when combined with rituximab and/or other chemotherapeutic agents in the treatment of lymphoid malignancies [9, 43–45]. Based on the pharmacokinetic characteristics of bendamustine and rituximab, a drug–drug interaction would not typically be expected. However, no formal clinical pharmacology study has been conducted to specifically assess pharmacokinetic interactions between bendamustine and other drugs. Furthermore, there are limited data on the pharmacokinetics of rituximab when combined with other drugs and on variables influencing individual exposure [46–48].

As noted before, bendamustine is a small molecule that is approximately 95 % bound to plasma proteins, mainly albumin [7, 19], and is primarily metabolized via hydrolysis [7, 14, 23]. In contrast, rituximab is a large molecule with targeted binding to CD20 antigen (but not to plasma proteins, such as albumin); a low volume of distribution (<3 L); and distinct elimination pathways that include hepatic proteolysis, the reticuloendothelial system, target-mediated elimination, and endocytosis [49–51]. However, the possibility of drug–drug interactions between bendamustine and rituximab cannot be completely ruled out. Indeed, indirect pharmacokinetic interactions between small molecules and monoclonal antibodies have been reported [48].

Two recent studies and data from the literature indicate that the potential for a drug–drug interaction between bendamustine and rituximab is low [52]. One of the studies was an open-label, multicenter, phase 3 study in adults who received bendamustine–rituximab combination therapy for advanced indolent NHL or mantle cell lymphoma [52]. The other study, which served as the data source for the bendamustine population pharmacokinetic model used in the combination therapy study, was the aforementioned phase 3 NHL study in adults who received bendamustine monotherapy [17].

### Bendamustine–rituximab combination therapy study

In the bendamustine–rituximab combination therapy study, patients received rituximab (375 mg/m^2^) followed by bendamustine (90 mg/m^2^) on day 1 of each cycle and bendamustine (90 mg/m^2^) on day 2 of each cycle. The final analysis dataset included bendamustine concentration samples from 49 patients and rituximab concentration samples from 19 patients [52].

### Bendamustine monotherapy study

In the bendamustine monotherapy study, patients received bendamustine (120 mg/m^2^) on days 1 and 2 of each cycle. Bendamustine plasma concentrations from 78 adult patients were described in a three-compartment, open population pharmacokinetic model with zero-order input and first-order elimination [17, 52].

### Effect of rituximab on bendamustine pharmacokinetics

Model-predicted Bayesian estimates of bendamustine clearance showed similar clearance values in patients who received bendamustine with or without rituximab, with median differences within 3.4 % of each other (32.9 vs. 31.8 L/h, respectively). The two groups did not differ significantly in their log-transformed clearance values (two-sided Wilcoxon signed rank test, *P* > 0.93) [52]. This finding is consistent with results of two small Japanese studies, in which the pharmacokinetic profile of bendamustine monotherapy was observed to be comparable with that of bendamustine in the presence of rituximab [28, 29].

### Effect of bendamustine on rituximab pharmacokinetics

Comparison between observed serum rituximab concentrations from the bendamustine–rituximab combination therapy study and those in four publications on the pharmacokinetics of rituximab (without bendamustine) in six different populations suggests that bendamustine does not affect the systemic exposure to rituximab [52–56]. For all studies, rituximab concentrations were compared at end of infusion (in the bendamustine–rituximab study, this was prior to bendamustine infusion, i.e., rituximab alone), 24 h, and 7 days post infusion. Median observed serum rituximab concentrations, in the absence (end of infusion) and presence of bendamustine (24 h and 7 days post infusion), in the bendamustine–rituximab combination therapy study were consistently lower than median weighted averages from literature data by 24, 30, and 53 % at each respective time point. However, the relative changes in serum concentrations over time were generally consistent across the studies [52–56]. Disparities in technique (e.g., duration of rituximab intravenous infusion, assay methodology, or assay sensitivity) could have resulted in the differences between the rituximab monotherapy findings across the studies [52].

## Conclusions

Maximal concentrations of bendamustine are typically reached at the end of infusion (~1 h), with rapid elimination characterized by an effective *t*_1/2_ of ~40 min and with no expected accumulation after multiple daily doses [7, 14, 17]. The compound is rapidly distributed to the site of action, but not extensively distributed into tissues [7, 17]. It primarily undergoes hydrolytic metabolism (without hepatic enzymes), into HP1 and HP2 metabolites, which have little or no activity [7, 14]. The active metabolites, M3 and M4, are formed via a hepatic CYP1A2 oxidative pathway [16]; however, their contributions to the cytotoxic effect of bendamustine is minimal [7, 16, 18]. In addition, renal elimination is minor; only ~3 % of a bendamustine dose is excreted in urine [18, 32].

Systemic exposure to bendamustine is similar in adults and pediatric patients [17, 27], which confirms the appropriateness of BSA-based dosing [27]. Age, sex, and race have minimal effects on the systemic exposure to bendamustine [7, 17, 27].

Bendamustine is not easily displaced by and does not displace other highly protein-bound drugs [19] and has a low likelihood of direct or indirect drug–drug interactions [52]. Based on in vitro and clinical data, *C*_max_ seems to be an important predictor of response to bendamustine [17, 42]. No clear dose–response relationship to efficacy has been observed, while higher doses may be associated with increased risk of nausea or infection [17, 27].

Bendamustine is a unique alkylator with demonstrated efficacy in NHL and CLL as well as clinical activity in a wide range of other malignancies [1–7]. Clinical experience with bendamustine has been extensive, and, together with its overall pharmacokinetics, pharmacokinetic/pharmacodynamic relationships, and drug–drug interactions, support the appropriateness of a BSA-based dosing scheme for a wide range of patient populations.
